# Intra-Individual Reaction Time Variability in Mild Cognitive Impairment and Alzheimer’s Disease: Gender, Processing Load and Speed Factors

**DOI:** 10.1371/journal.pone.0065712

**Published:** 2013-06-10

**Authors:** Michelle Phillips, Peter Rogers, Judy Haworth, Antony Bayer, Andrea Tales

**Affiliations:** 1 Avon and Wiltshire Mental Health Partnership NHS Trust, Research and Development Centre, Blackberry Hill Hospital, Bristol, United Kingdom; 2 School of Experimental Psychology, Bristol University, Bristol, United Kingdom; 3 Memory Disorders Clinic, Frenchay Hospital, Frenchay, Bristol, United Kingdom; 4 School of Medicine, University Hospital Llandough, Cardiff University, Wales, United Kingdom; 5 Department of Psychology, College of Human and Health Sciences, Swansea University, Wales, United Kingdom; University of Texas at Dallas, United States of America

## Abstract

Compared to cognitively healthy ageing (CH), intra-individual variability in reaction time (IIV_RT_), a behavioural marker of neurological integrity, is commonly reported to increase in both Alzheimer’s disease (AD) and mild cognitive impairment (MCI). It varies in MCI with respect to whether it represents the pro-dromal stages of dementia or not; being greatest in those most likely to convert. Abnormal IIV_RT_ in MCI therefore represents a potential measure of underlying functional integrity that may serve to differentiate MCI from CH and to help identify those patients for whom MCI is the result of a progressive pathological process. As the clinical approach to MCI is increasingly stratified with respect to gender, we investigated whether this factor could influence study outcome. The influence of RT_SPEED_ and processing load upon IIV_RT_ was also examined. Under low processing load conditions, IIV_RT_ was significantly increased in both MCI and AD compared to CH. However, correcting for an individual’s processing speed abolished this effect in MCI but not in AD, indicating that the increased IIV_RT_ in MCI and AD may result from different factors. In MCI but not in CH, IIV_RT_ was significantly greater for females. Increasing task processing load by adding distracting information, although increasing overall IIV_RT_, failed to improve the differentiation between CH and both MCI and AD, and in MCI resulted in a reduction in the influence of gender upon study outcome. The outcome of studies investigating IIV_RT_ in MCI and AD compared to CH therefore appear influenced by the gender of the participants, by task-related processing load and processing speed.

## Introduction

A behavioural measure of increasing interest in the study of mild cognitive impairment (MCI) and dementia is the intra-individual variability of reaction time (IIV_RT_) over the trials of a given task. This measure appears to be a behavioural indicator of neurological integrity, as a growing number of studies link IIV_RT_ to structural and functional brain characteristics. Indeed, DTI (diffusion tensor imaging) indicates a relationship between IIV_RT_ and white matter integrity, with increased variability indicative of white matter degradation, disconnectivity in associate pathways and brain dysfunction [1]–[11]. A wide range of behavioural studies now indicate changes in reaction time variability above and beyond slowing, with increased inconsistency linked with healthy ageing, impaired top-down executive and attentional control processes, cognitive disorder, neurotransmitter dysfunction, fatigue, stress [4], [6], [11]–[22] and various neurological, degenerative and psychiatric disorders including Parkinson’s disease [23]–[26], multiple sclerosis [27], schizophrenia [28] and brain injury [29], [30] and dementia [18]. Thus, behaviourally measured relative variability is a fruitful measure for the characterisation of healthy-ageing and pathological change.

Compared to cognitively healthy ageing (CH), IIV_RT_ is commonly reported to increase in both Alzheimer’s disease (AD) and MCI and to vary in MCI with respect to whether it represents the pro-dromal stages of dementia or not; being greatest in those most likely to convert [2], [3], [6]–[9], [12], [13], [15]–[17], [31]–[39]. Abnormal IIV_RT_ in MCI therefore represents a potential measure of underlying functional integrity that may serve to help identify those patients for whom MCI is the result of a progressive pathological process. Furthermore, the relationship between high IIV_RT_ and the breakdown in the integrity of information processing indicates its potential as an adjunct to neuropsychological assessment and the identification of those at risk of a greater degree of functional and behavioural impairment. However, although a measure of IIV_RT_ can be quickly and easily obtained under normal clinical conditions, investigation into the clinical applicability of IIV_RT_ is hindered by a degree of variability in study outcome and the interpretation of results (see [19] for a review). Although disparity in methodology and data analysis is a commonly posited causal factor, substantial individual differences in patients and controls also exist, both within and between studies. As a more individual or stratified approach is increasingly applied to the diagnosis and treatment of MCI [40] adopting a corresponding research approach to IIV_RT_ is therefore necessary to ensure outcome validity and relevance and it is the potential for some of these factors to affect study outcome that we explore in the present study. One of these factors is gender.

Gender is acknowledged as an important factor in clinical research in general and emerging evidence indicates that it may be of relevance with respect to the incidence, prevalence, risk profile, age-of-onset, effects, symptoms and severity of disease in MCI and AD e.g. [20], [40]–[46]. Many aspects of cognition and attention, together with some of the tests used for the diagnosis, staging and follow-up of MCI and AD, are also influenced by gender [40], [46]–[57]. White matter micro-architecture and cortico-cortical projections, with which the behavioural measure of IIV_RT_ is associated, may also be influenced by gender, although some of these differences appear to be site specific and/or inconsistent [1], [40], [43], [44], [46], [49], [52], [58], [59]–[65]. Such evidence increases the likelihood that clinically relevant gender-related factors arise in the investigation of IIV_RT_ in MCI, AD and CH. A common assumption in ageing, MCI and AD-related research is that any gender effects related to IIV_RT_ would be similarly expressed in patients and controls and, consequently, that simply ensuring similar ratios of males to females in the study groups would balance out any gender-related influence. In the present study we address this assumption by examining whether gender-related IIV_RT_ effects are expressed similarly in CH and MCI. In order to avoid potential confounds in the behavioural examination of gender related effects in IIV_RT_ in patients and controls it is imperative that the males and females within each group are matched with respect to cognitive function, diagnosis and demographic factors. Thus, in our study examining IIV_RT_ in CH and MCI the men and women within each group were matched as closely as possible in terms of age, pre-morbid IQ, MMSE score and z-score of a range of neuropsychological tests of memory, language, perception and executive function and diagnosis.

A contentious issue in this area of research is the relationship between processing or reaction time speed (RT_SPEED_) and IIV_RT_. Typically, RT_SPEED_ and IIV_RT_ are highly correlated to one another and in some instances raised IIV_RT_ appears to result simply from a correspondingly slowed RT_SPEED_ and debate continues with regards to whether IIV_RT_ that can be explained by slowing is clinically useful e.g. [2]. However, evidence that *both* RT_SPEED_ and IIV_RT_ are associated with neurological integrity, neurodegeneration, cognitive status and gender [1]–[3], [11], [19], [65]–[72] indicates that both measures may provide clinically relevant information. The fact that a significant group difference in IIV_RT_ might disappear when RT_SPEED_ is taken into account may simply indicate that, with respect to a particular task, the raised IIV_RT_ can be explained by RT slowing, but the fact remains that the slowing may itself be indicative of some degree of neurological disruption. To speculate further, whether or not slowed RT is the cause of increased IIV_RT_ in the study of MCI and dementia may be related to disease stage, i.e., a result of factors such as pathological burden, neurological and cognitive breakdown, aetiology and the presence or not of pro-dromal or frank dementia. A threshold of structural and functional integrity may exist: below which RT slowing is the main behaviourally observable change and the main contributory factor to an increase in IIV_RT_: above which, raised IIV_RT_ is the result of additional and possibly RT-independent neurological damage. We suggest therefore that in the absence of frank dementia, i.e., in MCI, raised IIV_RT_ is likely to be explained by concomitant slowing, whereas in AD, i.e., in dementia, it is not. Thus, in preliminary exploration of this idea, we examine IIV_RT_ with respect to RT _SPEED_ in both MCI and AD in the present study.

Whatever, the underlying cause, increased variability may still adversely affect information processing and thus behaviour and indicate the presence of neurological disruption. Nevertheless, whether or not adjustments are made for RT_SPEED_ can determine whether or not IIV_RT_ is reported as significantly greater in MCI and dementia than in CH [1], [11], [15], [19], [31], [33], [65] and whether gender related effects in IIV_RT_ are expressed or not [19], [49], [65]. Thus in the present study, we analyse both raw and RT_SPEED_-adjusted IIV_RT_ data in CH, MCI and AD in order to determine its effects upon study outcome and interpretation. Furthermore, although there is some evidence to suggest that it is IIV_RT_ rather than RT_SPEED_ that best differentiates both MCI and AD from CH, this is not always so [12], [15]–[17], [31], [32], [35] and RT_SPEED_ in its own right forms a substantial research area in ageing, MCI and dementia e.g. [12], [31], [73]–[75]. Consequently we also examine RT_SPEED_ per se in order to determine whether it is RT_SPEED_ or IIV_RT_ that results in the greatest group differentiation between CH and MCI and between CH and AD.

Methodologically, IIV_RT_ in ageing and dementia–related research has been examined using a wide range of paradigms. Following the general assumption that tasks with more complex or higher processing loads allow the accumulation of decline across multi-component processes [76] and are thus more likely to differentiate between CH and MCI and CH and AD, the majority of the tests used to measure IIV_RT_ have been described as having high processing, attentional or cognitive demands [2], [6], [12], [16], [17], [19], [65], [77]. However, such tests are difficult to compare and to quantify as they can vary not only in terms of the resources required to process the information contained in the RT task, but also with respect to the decision and motor components of the task response. Furthermore, simple tests can provide superior differentiation [6], [77]. It is likely therefore that the task used to examine IIV_RT_ has a substantial bearing on study outcome in clinical populations. To investigate this we employ a computer-based visual search task [78] in which the same decision and motor requirements for the target RT response are maintained under both low (target alone) and high (surrounding the target with distracting information) processing resource demands, see [31], [78]. The ‘target in isolation’ condition represents a typical computer-based visual choice RT task. Surrounding the same target with distracters of a similar form but differing orientation simulates the cluttered environment more typical in visual processing, in which more component processes, such as attention shifting, eye movements and the suppression or inhibition of irrelevant information, are required in order to find the target, thus slowing response time.

Debate also continues regarding which measure of intra-individual variability is used, i.e. standard deviation (SD) or inter-quartile range (IQR) (e.g. see [19] for a review) and which measure of processing speed is used, i.e. mean or median RT, and whether the inclusion or not of aberrant responses affects RT_SPEED_ and IIV_RT_. In order to examine these potential sources of study outcome variation, we measure individual RT_SPEED_ using both median and mean values and IIV_RT_ using both IQR and SD values with analysis performed both with and without the RTs responses for error trials.

To summarise, here we explore the potential influence of gender, RT_SPEED_, task processing demands, the unit of measurement and the inclusion or not of error responses, upon the study of IIV_RT_ in a group of patients highly typical of individuals presenting to memory clinics, namely those with amnestic multi-domain mild cognitive impairment (aMCI^+^). In a further study RT_SPEED_ and IIV_RT_ is examined in probable AD compared to CH. The relationship between RT_SPEED_ and IIV_RT_ in MCI and probable AD compared to cognitively healthy ageing is also examined.

## Study 1. Comparing IIV_RT_ in Amnestic Multi-domain Mild Cognitive Impairment and Cognitively Healthy Ageing

### Methods

#### Ethics statement

This study was conducted according to the principles in the Declaration of Helsinki. It was approved by Frenchay Research Ethics Committee and all participants gave written informed consent to participate. Only individuals with the capacity to consent were included. Capacity to consent was assessed by the clinician (JH) with specialist expertise in this field and consistent with the requirements of the Mental Capacity Act.

#### Participants

In line with the expected overall large effects for our study, based on previous research, the a-priori estimate of participant numbers was based on a statistical power level of.8, an anticipated effect size [Cohen’s *d*] of.7, and a probability level of.05, and revealed that, for two-tailed analysis, an approximate minimum total sample size of 68, with a minimum sample size per group of 34, was required.

Community dwelling cognitively healthy older adults (n = 62) and patients with aMCI^+^ (n = 55) were recruited through the Bristol Memory Disorders Clinic. All participants had normal or corrected-to-normal vision. Although medication could not be controlled in either group, none of the participants were receiving medication deemed likely to affect cognitive or attention-related function and none of the patients were receiving drug treatment or behavioural intervention of any kind for their cognitive dysfunction.

All participants performed a range of tests forming the typical Bristol Memory Disorders Clinic battery of neuropsychological tests that included MMSE [79], Wechsler Adult Intelligence Scale-III subtests [80], Hopkins Verbal Learning Test-Revised [81], CLOX [82], Visual Form Discrimination Task [83], National Adult Reading Test [NART [84]), S-word fluency and Animal fluency [85], Story Recall [Adult Memory Information Processing Battery [86], BADLS [87] and BASDEC (screen for depression) [88]. The CH adults had to perform at an age-appropriate level (z-score above −1.5) on all tests. All aMCI^+^ patients had self-reported change in memory, corroborated by an informant and objective decline, namely individual z-scores equal or less than −1.5 in memory and at least one other area of function, in the absence of dementia and an intact ability to perform activities of daily living (assessed using BADLS). Exclusion criteria included past history of serious head injury, stroke or other significant neurological or psychiatric condition. The clinical and demographic details for the CH and aMCI^+^ are shown in Table 1.

**Table 1:** Clinical and demographic details for the CH and aMCI^+^ groups.

	CH			aMCI^+^		
	Male (n = 31)	Female (n = 31)	All (n = 62)	Male (n = 26)	Female (n = 29)	All (n = 55)
**Age**	**70.5** (7.5)	**69.5** (8.5)	**69.9** (8.0)	**69.5** (7.2)	**67.4** (8.9)	**68.4** (8.1)
**NART**	**118.5** (7.8)	**118.1** (7.7)	**118.4** (7.6)	**112.8** (9.5)	**108.8** (11.1)	**110.6** (10.5)
**MMSE**	**27.2** (1.5)	**27.5** (1.5)	**27.3** (1.5)	**25.9** (1.6)	**25.9** (2.0)	**25.9** (1.8)

Mean age (in years), NART (predicted premorbid IQ) and MMSE score (total score/30) for the CH and aMCI+ groups by gender. Standard deviation (SD) in parenthesis.

The CH and aMCI^+^ groups did not differ significantly with respect to mean age [*t* (df 115) = 1.03, *p* = .31]. NART score was significantly poorer in the aMCI^+^ compared to the CH group [*t* (df^*^ 98.1) = 4.54, *p*<.001, effect size (Cohen’s *d)* = .77)] and, as to be expected, mean MMSE score was significantly lower in the aMCI^+^ compared to the CH group [*t* (df 115) = 4.51, *p*<.001]. Within the CH group, male and female participants did not differ with respect to mean age [*t* (df 60) = .44, *p* = .66], NART [*t* (df 60) = .19, *p* = .85] or MMSE, [*t* (df 60) = .77, *p* = .45]. Within the aMCI^+^ group, male and female participants did not differ with respect to mean age [*t* (df 53) = .95, *p* = .35], NART [*t* (df 53) = 1.43, *p* = .16] or MMSE [*t* (df 53) = .016, *p* = .99]. Note that here and throughout the manuscript df^*^ denotes the degrees of freedom correction used when equal variances cannot be assumed.

Within the aMCI^+^ group, z- scores for the neuropsychological tests mentioned in the clinical battery did not differ significantly with respect to gender [all *p*-values >.05], i.e., visual memory [*t* (df 53) = .37, *p = *.7], working memory [*t* (df 53) = 1.1, *p* = .27], immediate verbal memory [*t* (df 53) = .24, *p = *.8], delayed verbal memory [*t* (df 53) = 1.4, *p* = .17], verbal fluency [*t* (df 53) = .06, *p* = .9], semantic fluency [*t* (df 53) = 1.3, *p* = .2], CLOX [*t* (df 53) = .48, *p* = .63] or visual form discrimination [*t* (df 53) = .56, *p* = .58]. CH men however did show a significantly poorer z score for delayed verbal memory than women [*t* (df 57.3) = 2.4, *p = *.019 (equal variances not assumed)] but no other significant gender-related difference in neuropsychological test performance, i.e., no significant gender-related differences in visual memory [*t* (df 60) = .53, *p = *.6], working memory [*t* (df 60) = .24, *p* = .81], immediate verbal memory [*t* (df 60) = 1.6, *p* = .13], verbal fluency [*t* (df 60) = 1.5, *p* = .15], semantic fluency [*t* (df 60) = 1.27, *p* = .2], CLOX [*t* (df 60) = .36, *p* = .72] or visual form discrimination [*t* (df 60) = .88, *p* = .38].

#### Stimuli and tasks

**Figure 1:**
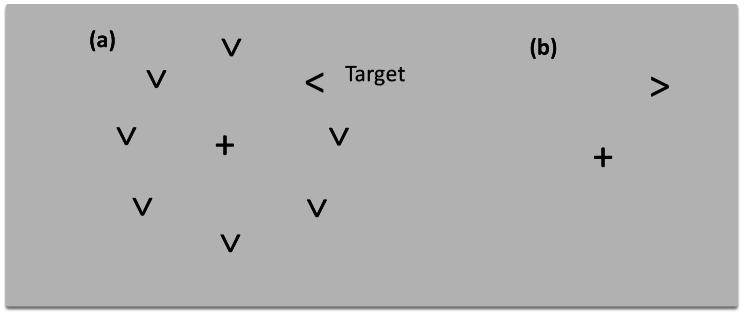
Representation of the stimuli used for (a) the target plus distracter condition and (b) the target alone condition.

Participants were asked to perform a simple computer-based visual search task and one used in several previous studies e.g. [70] in which the time taken to respond to a target (target discrimination) when it appeared in isolation upon the screen and the time taken to respond to the same target when it was surrounded by similar but irrelevant and distracting stimuli was determined. This paradigm [78] was presented on a Toshiba Satellite-Pro laptop computer viewed at a distance of 57 cm. Stimulus presentation and response recording was performed using Superlab software (Cedrus Corporation San Pedro, CA). All trials included a black target that was either a left or right-pointing arrow, i.e., a choice RT task. The task was to indicate whether the arrow was pointing to the right or left. The distracting stimuli consisted of seven black arrows that pointed up and down. A ‘clock-face’ configuration (see Fig. 1) was used to position the target, both when it appeared alone and when surrounded by 7 distracters, in a specific counterbalanced arrangement in order to eliminate any differences in processing between right and left and upper and lower visual fields. A total of 64 trials were presented; the target appearing 8 times at each of the possible ‘clock-face’ locations. For one half of the trials distracters were presented at the other locations and for the other half no distracters were presented. For each trial the central fixation cross appeared on screen for 1000 ms prior to the appearance of the target (with or without distracters) and remained on screen for the duration of the trial. The stimuli remained on screen until the participant responded, after which the fixation point appeared again. The participants were instructed to fixate on the centre cross at the beginning of each trial and to respond as quickly but as accurately as possible as to whether the target was pointing to the right or left by pressing one of two computer keyboard keys. After instruction, all participants were asked to explain the task to the researcher in order to demonstrate that they fully understood the requirements of the task and then to perform a practice block of approximately 10 trials. The ability of the participant to fixate on the cross at the beginning of each trial continued to be checked throughout the procedure by researcher observation. The participants received no feedback about their performance during the test [78].

Group mean analysis for RT_SPEED_ was based on both the median and mean values for each individual within the group. Likewise, group mean analysis for IIV_RT_ was based on both the IQR [between 75^th^ and 25^th^ quartiles] and SD values for each individual within the group using data both including and excluding error RT responses. However, to pre-empt our results, the inclusion or exclusion of error RTs, the use of mean or median measures of RT_SPEED_ and the use of SD or IQR measures of IIV_RT_, did not alter study outcome, with RT_SPEED_ and IIV_RT_ remaining highly correlated to one another irrespective of how they were measured. Therefore, we report only the results for error-excluded median-based RT_SPEED_ and IQR-based IIV_RT_ analysis. Parametric statistical analysis was applied to the data, with corrections made for conditions under which equal variances could not be assumed. To ensure a robust statistical approach non-parametric analysis was also applied to the data but this resulted in no change in study outcome, thus in line with common practice we report the parametric analysis.

The RT_SPEED_, IIV_RT_ and the coefficient of IIV_RT_ in response to the target appearing in isolation constituted the low processing condition and can be seen in Table 2. For the increased processing load condition, the target alone RT_SPEED_, IIV_RT_ and the coefficient of IIV_RT_ values were subtracted from those for the target plus distracters condition [target plus distracters – target alone], see Table 3. All analysis was performed at the two-tailed level.

**Table 2:** Data for each sample group under low processing load conditions.

	CH			aMCI^+^		
	Male (n = 31)	Female (n = 31)	All (n = 62)	Male (n = 26)	Female (n = 29)	All (n = 55)
**RT speed**	**738.4** (159.3)	**757.2** (153.2)	**747.8** (155.3)	**834.6** (177.8)	**1040.5** (394.8)	**943.2** (325.9)
**IIV_RT_ (IQR)**	**220.7** (102.2)	**210.5** (85.2)	**215.6** (93.5)	**235.6** (98.5)	**403.5** (292.3)	**324.1** (236.6)
**IIV_RT_ (Coef)**	**29.3** (10.6)	**27.4** (8.3)	**28.4** (9.5)	**28.1** (9.1)	**37.2** (20.7)	**32.9** (16.8)
**% errors**	**1.9**%	**2.6**%	**2.3**%	**2.9**%	**5.0**%	**4.0**%

Group mean RT_SPEED_ (msec) derived from individual median values, group mean IIV_RT_ derived from individual IQR values, their corresponding coefficient (Coef) values and the percentage of excluded trials for the CH and aMCI^+^ groups (standard deviation in parenthesis).

**Table 3:** Data for each sample group.

	CH			aMCI^+^		
	Male (n = 31)	Female (n = 31)	All (n = 62)	Male (n = 26)	Female (n = 29)	All (n = 55)
**RT speed**	**923.7** (295.7)	**1049.5** (490)	**986.6** (406.3)	**1315.2** (501.1)	**1649.3** (851.7)	**1491.3** (721.6)
**IIV_RT_ (IQR)**	**867.9** (376.5)	**1072.0 (**680.5)	**970.0** (555.0)	**1162.7** (504.8)	**1369.5** (745.0)	**1271.7** (645.4)
**IIV_RT_ Coef**	**35.1** (17.9)	**43.3** (26.8)	**39.2** (23.0)	**37.1** (17.6)	**29.6** (32.4)	**33.2** (26.5)
**% errors**	**−1.3**%	**−0.3%**	**−0.8%**	**−0.3%**	**−0.2%**	**−0.3%**

The difference in RT speed and intra-individual variability between the high and low processing load conditions.

The difference [target plus distracters – target alone] in RT_SPEED_, (msec), IIV_RT_ and corresponding IIV_RT_ coefficient and percentage errors (standard deviation in parenthesis).

### Results

#### Low processing load conditions

**Figure 2:**
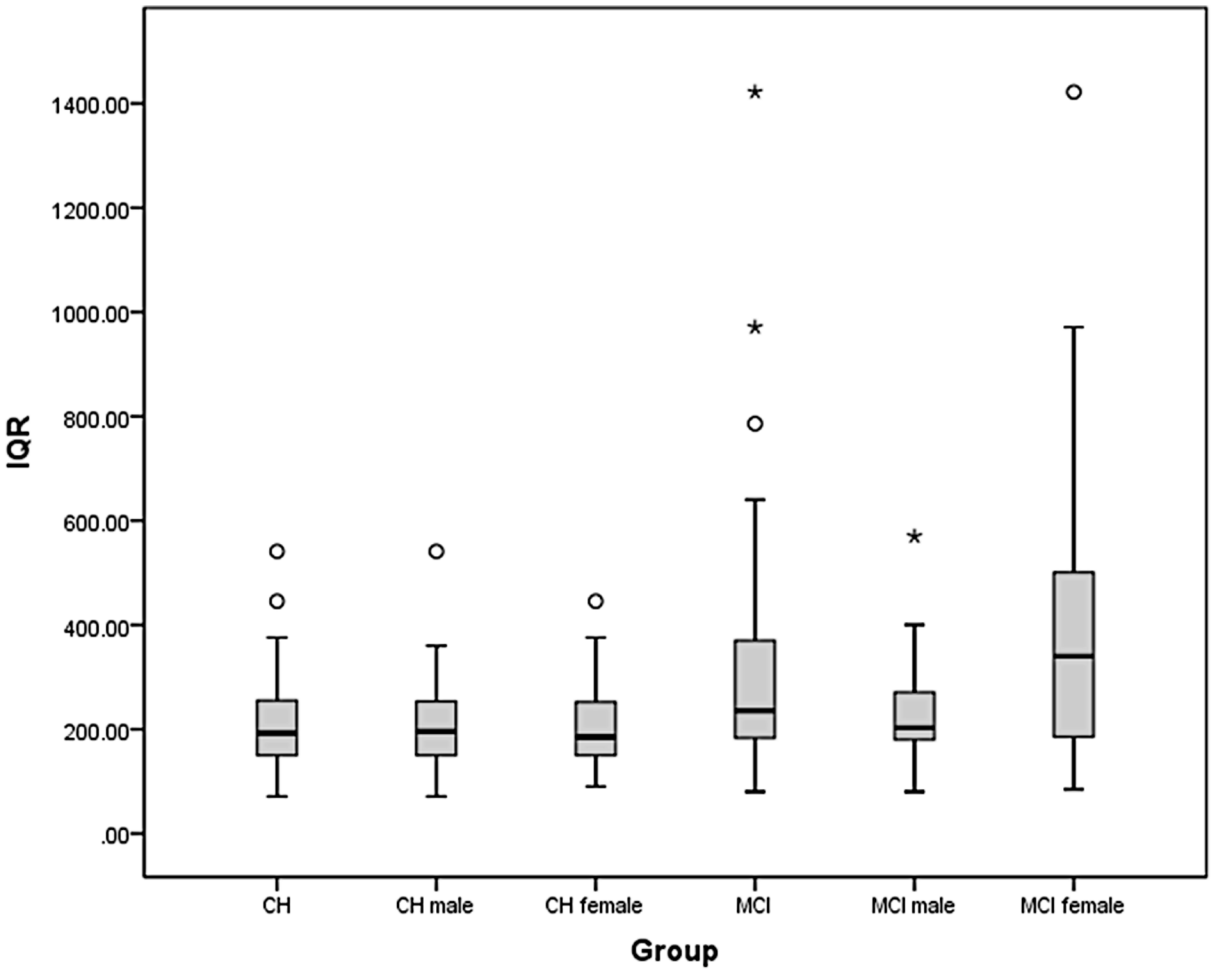
Box plot of IIV_RT_ (msec) based on individual IQR values for the cognitively healthy older adult controls (Old) and patients with aMCI ^+^(MCI).

**Figure 3:**
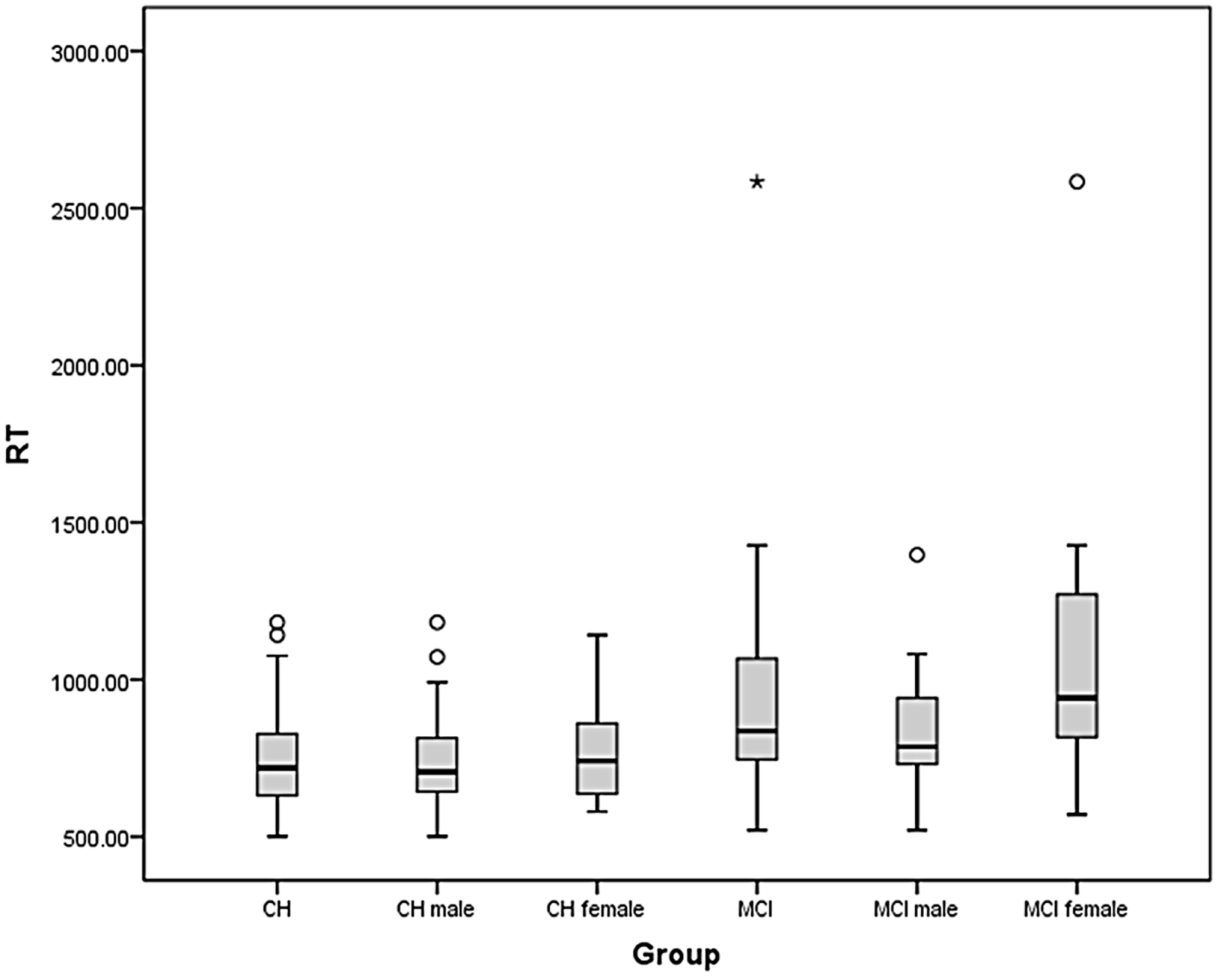
Box plot of the RT_SPEED_ (msec) for the cognitively healthy older adult controls (Old) and patients with aMCI^+^ (MCI).

Under low processing load conditions, the box-plots (Figures 2 and 3) and Table 2 reveal a greater degree of IIV_RT_ and slower RT_SPEED_ for the aMCI^+^ compared to the CH group. Pronounced gender-related effects within the aMCI^+^ group, together with greater within-group variability in IIV_RT_ and RT_SPEED_ in aMCI^+^ compared to cognitively healthy ageing are also evident. Group mean RT_SPEED_, was significantly slower in aMCI^+^ compared to CH [*t* (df*75.2) = 4.06 *p*<.001, effect size (Cohen’s *d*) = .78)]. For the CH group, RT_SPEED_ was significantly correlated with age [*r* = .27, *p* = .037], but not with NART [*r* = −.114, *p* = .38], or MMSE [*r* = −.117, *p* = .36]. The same analysis for the aMCI^+^ group revealed that RT_SPEED_ was not significantly correlated with RT [*r* = .039, *p* = .78], MMSE [*r* = .021, *p* = .88] or age [*r* = .054, *p* = .7].

Group mean IIV_RT_ was significantly greater in aMCI^+^ compared to CH [*t* (df^*^ 68.8) = 3.19, *p* = .002, effect size (Cohen’s *d*) = .63)]. For the CH group IIV_RT_ was not significantly correlated with NART [*r* = .08, *p* = .53], MMSE [*r* = −.063, *p* = .63] or age [*r* = .14, *p* = .28] and that similarly, for the aMCI^+^ group, IIV_RT_ was not significantly correlated with NART [*r* = .056, *p = *.7], MMSE [*r* = −.075, *p* = .6] or age [*r* = .014, *p* = .92].

Converting the IQR measure of IIV_RT_ to its coefficient, i.e., [(IQR/median RT_SPEED_) ×100] eliminated the significantly greater IIV_RT_ in aMCI^+^ compared to CH [*t* (df^*^ 83) = 1.78, *p = *.079]. The mean percentage of errors was low overall and did not vary significantly between CH and aMCI^+^ [*t* (df^*^ 96.6) = 1.87, *p* = .07].

With thanks to anonymous reviewers for suggesting further analysis, we examined RT_SPEED_ and IIV_RT_ with respect to the neuropsychology test z scores for both groups. For the CH group, RT_SPEED_ was not significantly correlated with performance on any of the tests [all *p*-values >.05] and IIV_RT_ was found to be significantly correlated only with semantic fluency performance [*r* = −.33, *p* = .01]. For the aMCI^+^ group both RT_SPEED_ and IIV_RT_ were significantly correlated with performance on the Visual Form Discrimination Task [*r* = −.27, *p* = .045] and [*r* = −.3, *p = *.026] respectively, but not with the performance of any of the other neuropsychological tests. Note also that the significant outcomes do not survive Bonferroni correction.

#### Gender

In CH, neither IIV_RT_ or RT_SPEED_ differed significantly with respect to gender [*t* (df 60) = .43, *p* = .67] and [*t* (df 60) = .47, *p* = .64] respectively. For the females in the CH group, RT_SPEED_ was not significantly correlated with NART [*r* = −.045, *p* = .81], MMSE [*r* = −.22, *p* = .25] or age [*r* = .26, p = .17] and likewise, IIV_RT_ was not significantly correlated with NART [*r* = .047, *p* = .8], MMSE [*r* = .03, *p* = .87] or age [*r* = .03, *p* = .87]. We examined correlations between RT_SPEED_ and IIV_RT_ and neuropsychology test z scores separately for females and males and for both groups. For the females, neither RT_SPEED_ or IIV_RT_ was significantly correlated to any neuropsychology test score [all *p*-values >.05]. The same analysis for the males within the CH group also revealed that RT_SPEED_ was not significantly correlated with NART [*r* = −.18, *p* = .34], MMSE [*r* = −.041, *p* = .83] or age [*r* = .29, *p* = .12] and likewise that IIV_RT_ was not significantly correlated with NART [*r = *.11, *p* = .56], MMSE [*r* = −.13, p = .49] or age [*r = *.24, *p* = .19]. However, with respect to neuropsychological test z score for males, RT_SPEED_ was significantly correlated with semantic fluency [*r* = −.41, *p* = .022] and IIV_RT_ was significantly correlated with semantic fluency [*r* = −.421, *p* = .018] and with CLOX score [*r* = −.5, *p* = .004]. Note however, that only the significant outcome for the CLOX analysis survived Bonferroni correction.

In contrast, for the aMCI^+^ group, mean IIV_RT_ was significantly greater in females compared to males [*t* (df^*^ 34.9) = 2.92, *p = *.006, effect size (Cohen’s *d*) = .8)] with RT_SPEED_ significantly slower for female than male patients [*t* (df^*^ 39.8) = 2.54, *p* = .015 effect size (Cohen’s *d*) = .7)]. For the female patients, RT_SPEED_ was not significantly correlated with NART [*r* = .23, *p = *.23], MMSE [*r* = −.025, *p* = .9] or age [*r* = .09, *p* = .64] and similarly IIV_RT_ was not significantly correlated with NART [*r = *.256, *p* = .2], MMSE [*r* = −.13, *p* = .52] or age [*r = *.034, *p* = .86]. Furthermore, for the female patients neither RT_SPEED_ or IIV_RT_ performance was significantly correlated to any neuropsychological test z score. For the male patients, RT_SPEED_ was not significantly correlated with NART [*r* = −.23, *p* = .26], MMSE [*r* = .17, p = .40] or age [*r* = .14, p = .48] and likewise, IIV_RT_ was not significantly correlated with NART [*r* = −.24, *p* = .23], MMSE [*r = *.083, *p* = .7] or age [*r* = .22, *p* = .29]. Furthermore, for the male patients, RT_SPEED_ was significantly correlated with immediate verbal memory [*r* = .523, *p* = .006] but with no other neuropsychological test score [all *p*-values >.05].

In the CH group converting IQR to its coefficient value [*t* (df ^*^56.8) = .79, *p* = .43 did not alter the lack of a gender effect. However, in the aMCI^+^ group converting IQR to its coefficient value eliminated the significantly greater IIV_RT_ for females compared to males [*t* (df^*^ 44.7) = 1.6, *p* = .12].

#### Increasing the processing load

Raising the processing load by surrounding the target with distracters resulted in significantly slowed mean RT_SPEED_, compared to that evoked by the target in isolation for both the CH [*t* (df 61) = 19.1, *p*<.001] and aMCI^+^ [*t* (df 54) = 15.3, *p*<.001] groups although the magnitude of this effect was significantly greater for the aMCI^+^ compared to the CH group [*t* (df^*^ 82.85) = 4.58, *p*<.001, effect size (Cohen’s *d*)* = *.9].

Increasing the processing load also resulted in a significantly increased IIV_RT_ for both the CH [*t* (df 61) = 13.76, *p*<.001] and aMCI^+^ [*t* (df 54) = 14.6, *p*<.001] groups respectively compared to that evoked for the target in isolation. The magnitude of this effect was however significantly greater for the aMCI^+^ compared to the CH group [t (df^*^ 107.2) = 2.7, *p* = .008.effect size (Cohen’s *d*) = .52]. Converting IQR to its coefficient value eliminated the significantly greater IIV_RT_ for the aMCI^+^ compared to the CH group [*t* (df 115) = 1.33, *p* = .19].

The distracter-induced increase in mean RT_SPEED_ did not differ significantly between males and females in the CH [*t* (df 60) = 1.46, *p* = .15] or the aMCI^+^ group [*t* (df 53) = 1.75, *p* = .09] respectively. Similarly, the distracter-induced increase in mean IIV_RT_ did not differ with respect to gender in the CH [*t* (df 60) = 1.46, *p* = .15] or the aMCI^+^ group [*t* (df 53) = 1.75, *p* = .087] respectively. For CH, converting IQR to its coefficient value did not alter the effect [*t* (df 60) = 1.41, *p* = .16] between males and females. For the aMCI^+^ group converting IQR to its coefficient value did not alter the effect [*t* (df ^*^44.2) = 1.1, *p* = .28] between males and females.

#### Errors

For the low processing load condition the mean percentage of errors was very low for both the CH and the aMCI^+^ groups and adding distracters actually resulted in an overall, but not significant, reduction in the mean percentage of errors made (denoted by the −% value in table 3) for the CH [*t* (df = 61) = 1.36, *p* = .18] and for the MCI [*t* (df 54) = .39, *p* = .7] groups. As is evident from Tables 2 and 3 the mean percentage change in errors also did not vary significantly with respect to group, gender or task. And indeed as already highlighted the inclusion or exclusion of error-related data did not affect study outcome.

## Study 2: Comparing RT_SPEED_ and IIV_RT_ in Alzheimer’s Disease and Cognitively Healthy Ageing

As described in the introduction, several previous studies comparing probable AD to CH have shown significantly raised RT_SPEED_ and significantly slower IIV_RT_ in probable Alzheimer’s disease compared to CH. However, as in aMCI^+^, variability in AD-related study outcome exists, particularly with respect to how IIV_RT_ is measured. In view of the importance of replicability in research we examine the status of RT_SPEED_ and IIV_RT_ in AD compared to CH. As in our study of aMCI^+^, group mean analysis for RT_SPEED_ was based on both the median and mean values for each individual within the group. Similarly, group mean analysis for IIV_RT_ was based on both the IQR [between 75^th^ and 25^th^ quartiles] and SD values for each individual within the group, using data both including and excluding error RT responses. However, to pre-empt our results, the inclusion or exclusion of error RTs, the use of mean or median measures of RT_SPEED_ and the use of SD or IQR measures of IIV_RT_, did not alter study outcome, with RT_SPEED_ and IIV_RT_ remaining highly correlated to one another irrespective of how they were measured. Therefore, we report only the results for error-excluded median-based RT_SPEED_ and IQR-based IIV_RT_ analysis. Furthermore, in the introduction we suggested that slowed RT would explain the increased IIV_RT_ in aMCI^+^ but not in AD. In our study of aMCI^+^ and CH described earlier, we found that the raised IIV_RT_ in aMCI^+^ compared to CH could be accounted for by a slowing in RT_SPEED_. In the following study we examined whether the increased IIV_RT_ in AD could be accounted for by a slowing in RT_SPEED_.

### Participants

We predicted a significantly greater RT_SPEED_ and IIV_RT_ in AD compared to CH and at levels greater than that seen in aMCI^+^, thus a-priori power analysis estimation was based upon one-tailed analysis with an estimated effect size of at least.9, a statistical power level of.8 and a probability level of.05, giving a required minimum total sample group of 32 participants. Community dwelling cognitively healthy older adults (n = 17; 9 males, 8 females) and patients with probable AD (n = 17; 7 males, 10 females) were recruited through the Bristol Memory Disorders Clinic and tested. AD was diagnosed with respect to standard clinical criteria [89] using the same investigations in the previously described study of aMCI^+^ patients. The controls were also assessed using this same procedure. Although medication could not be controlled in either group, none of the participants were receiving medication deemed likely to affect cognitive or attention-related function and none of the patients were receiving drug treatment or behavioural intervention for AD at the time of testing. Exclusion criteria included past history of serious head injury, stroke or other significant neurological or psychiatric condition. The task and procedure were identical to those used in the study of the aMCI^+^ group. The clinical and demographic details for the CH and AD groups are shown in Table 4. The RT_SPEED_ and IIV_RT_ data for these two groups are shown in Table 5.

**Table 4:** Clinical and demographic details for the CH and AD groups.

	CH (n = 17)	AD (n = 17)
**Age**	**76.7** (5.5)	**78.4** (7.9)
**NART**	**119.9** (9.3)	**107.2** (9.6)
**MMSE**	**26.7** (1.7)	**18.9** (2.7)

Mean (SD) age (years), NART (predicted pre-morbid IQ) and MMSE score total score/30 for the CH and AD groups.

**Table 5:** RT and IIV _RT_ data for each sample group.

	TARGET ALONE		DIFFERENCE	
	CH (n = 17)	AD (n = 17)	CH (n = 17)	AD (n = 17)
**RT speed**	**773.5** (156.5)	**1748.1** (834.4)	**910.1** (231.7)	**4387.6** (3724.4)
**IIV_RT_ (IQR)**	**214.5** (66.9)	**1147.6** (1013.0)	**1009.7** (352.9)	**3900.2** (3419.6)
**IIV_RT_ Coef**	**27.4** (4.6)	**57.7** (24.6)	**45.6** (19.7)	**23.5** (34.8)
**% errors**	**1.1**%	**7.91**%	**−.55%**	**−.54%**

Group mean target alone values and difference values [target plus distracters – target alone] for RT_SPEED_ (msec), group mean IIV_RT_, their corresponding coefficient values and the percentage of excluded trials for the CH and AD groups (standard deviation in parenthesis).

Although age did not vary significantly between the two groups [*t* (df^*^ 28.6) = .71, *p* = .49], both NART and MMSE were significantly poorer in AD than CH (*t* (df 32) = 3.95, *p*<.001, effect size (Cohen’s *d*) = 1.4)] and [*t* (df 32) = 10.17, *p*<.001, effect size (Cohen’s *d*)* = *3.5)] respectively.

Two-tailed analysis revealed that RT_SPEED_ was significantly slower in the group of patients with AD compared to the CH group [*t* (df ^*^17.2) = 4.74, *p* = <.001, effect size (Cohen’s *d*) = 1.62]. IIV_RT_ was significantly greater in AD compared to CH [t (df^*^ 16.1) = 3.79, *p = *.002, effect size (Cohen’s *d*)* = *1.3]. Converting IQR to its coefficient value still revealed a significantly greater IIV_RT_ in AD than CH [*t* (df ^*^17.1) = 5.0, *p*<.001, effect size (Cohen’s *d*) = 1.71). The AD group displayed a significantly greater percentage of errors compared to the CH group [*t* (df^*^ 17.14) = 3.39, *p* = .003 (equal variances not assumed)]. However, the inclusion of error related data or not did not affect study outcome.

### Increasing the Processing Load

For the CH and AD groups, raising the processing load by surrounding the target with distracting information resulted in a significant slowing in RT speed [*t* (df 16) = 16.18, *p*<.001 and [*t* (df 16) = 5.5, *p*<.001] respectively but RT slowing was significantly greater for the AD compared to the CH group [*t* (df^*^ 16.1) = 3.84, *p* = .001, effect size (Cohen’s *d*) = 1.4].

For both CH and AD groups, adding distracters also led to a significant increase in IIV_RT_ [*t* (df 16) = 11.8, *p*<.001] and [*t* (df 16) = 4.7, *p*<.001] respectively. The magnitude of the increase in IIV_RT_ in response to the distracting information was significantly greater for the AD compared to the CH group [*t* (df^*^ 16.3) = 3.46, *p* = .003, effect size (Cohen’s *d*) = 1.2)]. Converting IQR to its coefficient in both the CH and AD group still revealed a significantly greater IIV_RT_ for the addition of distracters [*t* (df 16) = 9.5, *p*<.001] and [*t* (df 16) = 2.8, *p = *.013] respectively. Converting IQR to its coefficient value still showed a significantly greater IIV_RT_ in AD than CH [*t* (df^*^ 25.3) = 2.3, *p* = .032, effect size (Cohen’s *d*) = .8)]. Adding distracting information reduced the percentage of errors for the CH and AD groups, but the reduction in AD was significantly greater compared to CH [*t* (df 17.2) = 3.4, *p* = .003] although again, whether error-related RT and IIV was included in analysis or not did not affect study outcome.

### Discussion

We investigated the potential influence of RT_SPEED_, gender, task processing demands, the form of measurement (SD or IQR), and the inclusion or not of error responses, upon the study of IIV_RT_ in aMCI^+^ compared to CH. The relationship between RT_SPEED_ and IIV_RT_ in aMCI^+^ and AD compared to CH was also investigated. In brief, the unit of measurement for both RT_SPEED_ and IIV_RT_, and whether error response RTs were included in the analysis or not, failed to alter study outcome. In contrast, gender, processing load and whether RT_SPEED_ was taken into account in the statistical analysis did influence the results. Furthermore, RT_SPEED_ appeared to account for the raised IIV_RT_ in aMCI^+^ but not in AD. In the following sections we discuss these findings in greater detail.

### Low Task Processing Load Conditions

When the target appeared in isolation, group mean RT_SPEED_ was significantly slowed and IIV_RT_ significantly raised in the both aMCI^+^ and AD groups compared to their respective CH control group; an outcome typical of many previous studies [16], [17], [31], [73], [74]. Although no direct statistical comparison was performed, compared to CH, the slowing of RT_SPEED_ was more pronounced in AD, (effect size (Cohen’s *d*) = 1.62) than in aMCI^+^ (effect size (Cohen’s *d*)* = *.78) and the increase in IIV_RT_ were more pronounced in AD (effect size (Cohen’s *d*) = 1.3) than in aMCI^+^ (effect size (Cohen’s *d*)* = *.63) compared to CH. This, together with evidence from previous studies (showing that RT_SPEED_ and IIV_RT_ can be preserved in amnestic MCI (aMCI) [e.g.35]) indicates that both measures are sensitive to the degree of cognitive decline, pathological load and neurological dysfunction [34], [90] all of which might be expected to be greater in aMCI^+^ than in aMCI, or in the presence of prodromal and frank dementia.

In both the CH and aMCI^+^ groups, (for which we had specific neuropsychological test data) neither RT_SPEED_ or IIV_RT_ was significantly correlated with MMSE, NART or neuropsychological (z-score) performance. This may indicate that RT_SPEED_ and IIV_RT_ are influenced by disease-related factors largely independent of those influencing cognitive performance and cognitive reserve and the possibility arises that in aMCI^+^, and indeed in AD, the deficits in IIV_RT_ and RT_SPEED_ occur in parallel to but are not directly related to changes in cognition and underlying cognitive reserve. Alternatively, as our study was not designed primarily to investigate such function with respect to RT_SPEED_, IIV_RT_ and the coefficient of IIV_RT_ in CH, aMCI^+^ and AD, such analysis may be under-powered leading to the expression of a Type II error, rendering further investigation imperative.

As in previous reports e.g. [2] IIV_RT_ was significantly positively correlated with RT_SPEED_ in CH, aMCI^+^ and AD. Further analysis using coefficient values of IQR, revealed that IIV_RT_ was no longer significantly increased in aMCI^+^ compared to CH when RT_SPEED_ was taken into account, whereas it remained significantly increased in AD compared to CH. These results indicate that the outcome of studies of IIV_RT_ in MCI, i.e., whether IIV_RT_ is significantly different in MCI compared to CH or not, can vary with respect to whether RT_SPEED_ is taken into account in between-group analysis, a finding in accord with several previous studies e.g. [1], [11], [15]–[17], [19], [31], [38], [65]. As predicted, our results also reveal that although slowed RT_SPEED_ accounts for the greater IIV_RT_ in aMCI compared to CH, it does not account for the significantly raised IIV_RT_ found in AD compared to CH. To speculate, disease burden may be so great in AD compared to aMCI^+^ that it actually interferes with or changes processing, rather than simply slowing it. However, as highlighted by one of our anonymous reviewers, it is possible that aMCI^+^ and AD differ with respect to whether or not RT_SPEED_ accounts for raised IIV_RT_ as a result of a relative lack of power in one of the studies. Thus future studies, with larger and more similar sample sizes, would more confidently confirm such results.

Furthermore, although there is some evidence from previous studies to suggest that it is IIV_RT_ rather than RT_SPEED_ that better differentiates MCI from CH [12], [15]–[17], [31], [32] our results reveal a slightly greater effect size when RT_SPEED_ rather than IIV_RT_ is used (Cohen’s *d* = .78) and (Cohen’s *d* = .63) respectively. It is likely therefore study outcome is dependent upon methodology and group demographics and disease factors.

Given the relationship between both slowed RT_SPEED_ and raised IIV_RT_ and impaired neurological integrity it is not surprising that IIV_RT_ was significantly increased and RT_SPEED_ significantly slowed in aMCI^+^ compared to CH. However, to what degree this difference in aMCI^+^ can be explained by the presence of prodromal dementia in a proportion of the aMCI^+^ group or the result of cognitive change per se remains to be determined using longitudinal methodology. Another point to consider in relation to cognitive function relates to cognitive reserve. In the present study, NART score, (a measure of pre-morbid IQ and one which is often used as a proxy for cognitive reserve), was significantly lower in aMCI^+^ compared to CH. This indicates that the patient group may have had lower pre-morbid IQ and thus lower cognitive reserve per se compared to the CH group. It is possible therefore that the significant reduction in RT_SPEED_ and IIV_RT_ in aMCI^+^ is the result, at least in part, of differences in pre-morbid IQ and cognitive reserve. However, as discussed earlier, this lack of a significant relationship between pre-morbid IQ (NART; cognitive reserve), cognition, MMSE score and RT_SPEED_ and IIV_RT_, may represent the expression of Type II error occurring in the absence of an appropriately powered study designed to look specifically at these factors. Nevertheless, whatever the underlying cause of the difference in IIV_RT_ and RT, and whatever the link between MMSE, cognition and pre-morbid IQ or cognitive reserve and IIV_RT_ and RT speed and actual brain structure and function, our results indicate a greater degree of disruption to information processing in aMCI^+^ than in CH.

### Gender

In CH, mean RT_SPEED_ and IIV_RT_ did not differ significantly between men and women. In aMCI^+^, RT_SPEED_ was significantly slower, and IIV_RT_ significantly greater, in female compared to male patients, although this effect was abolished when RT_SPEED_ was taken into account. The raised IIV_RT_ in female patients therefore appeared to be the result of their greater degree of slowing compared to the male patients. Nevertheless, given the relationship between both slowed RT_SPEED_ and raised IIV_RT_ and impaired neurological integrity, this finding still indicates a greater degree of neurological dysfunction in the female patients, a finding in support of some neuroimaging and pathological studies e.g. [57], [91], [92]. Furthermore, the significant female-related increase in IIV_RT_ and slowing of RT_SPEED_ compared to men seems to be a disease-rather than a normal ageing-related effect. However, some caution in interpreting this outcome is necessary. One should note that within the aMCI^+^ group, despite the large effect size (Cohen’s *d*) of.8 for the gender difference in IIV_RT_ and the large effect size (Cohen’s *d*) of.7 for the gender difference in RT_SPEED_, this aspect of the study may have been relatively underpowered as the numbers of males and females within the CH group were relatively low. Clearly therefore further study is required with increased participant numbers. Nevertheless, this preliminary indicator of potentially different gender-related influences upon RT_SPEED_ and IIV_RT_ in aMCI^+^ and CH indicates (as discussed in the introduction) that it may not be appropriate to assume that in ageing, MCI and AD-related research, any gender effects related to IIV_RT_ are similarly expressed in patients and controls.

Although it was not possible to verify the relationship between white matter integrity, cognitive function, pre-morbid IQ (cognitive reserve) and IIV_RT_ and RT function in the present study, the female patients with aMCI^+^ appeared able to perform at a similar cognitive level as male patients despite evidence from RT_SPEED_ and IIV_RT_ measures of a greater underlying neurological dysfunction. One could argue therefore that this provides evidence for a greater degree of cognitive reserve in female compared to male patients, (a finding in accord with some previous studies). Although our NART proxy measure of cognitive reserve does not support this hypothesis, it is possible that this results from the fact that our study was not specifically designed, and thus powered, to study gender-related cognitive reserve as measured by NART proxy. Clearly, however, the evidence for such gender-related discrepancy indicates that further research is required in order to determine the relationship between white matter integrity, IIV_RT_ and RT function, cognitive performance and cognitive reserve and gender.

In view of evidence showing that for MCI the risk of progression to dementia is greater in females than males and that females may progress more rapidly through the transition phase to AD [42], it is also possible that IIV_RT_ was greater in female than male aMCI^+^ patients in our study because they were more likely to have prodromal dementia, or simply at a later disease stage, despite our gender-matching on behavioural measures of cognitive function, MMSE, diagnosis and stage. In the absence of longitudinal follow up, this possibility cannot be determined in the present study. Nevertheless, irrespective of causality, our results indicate that in patients newly diagnosed with aMCI+ (as were ours), females can exhibit a similar cognitive and diagnostic profile to men but in fact be suffering considerably worse neurological disruption, which although not ostensibly affecting clinical measures of cognition, may have a detrimental impact upon other aspects of brain processing and thus behaviour. This may be particularly important when one considers the evidence to suggest that those with greater reserve are less amenable to early detection using cognitive measures [93]. However, one must apply some caution to such speculation until studies with greater numbers of male and female participants can be performed.

Our results provide additional evidence to support the consideration of gender stratification in research and in the interpretation of results in clinical practice. The importance of considering the influence of gender upon study outcome when investigating RT_SPEED_ and IIV_RT_ in MCI and AD compared to CH is also confirmed by these results.

### Increasing the Processing Load

As expected, raising the processing load by surrounding the target with distracting information slowed mean RT_SPEED_ and raised IIV_RT_ compared to that evoked by the low processing load task in CH, aMCI^+^ and AD, with the effect being significantly greater in both patient groups compared to CH. This indicates that task variation, particularly in processing load, may be a factor to consider when examining outcome variation in such studies.

When RT_SPEED_ was taken into account the significantly greater IIV_RT_ in aMCI^+^ compared to CH was abolished, thus indicating that, as in the case for the low processing load condition, raised IIV_RT_ in aMCI^+^ compared to CH can be explained by their slowed RT_SPEED_. In contrast, when RT_SPEED_ was taken into account in the comparison of IIV_RT_ in AD compared to CH under high processing load conditions the significantly greater IIV_RT_ in AD was replaced by a significantly greater IIV_RT_ in CH compared to AD. This indicates that RT_SPEED_ explains the greater IIV_RT_ in AD compared to CH under high processing load conditions and that, in CH, raising the processing load can increase IIV_RT_ independently of RT_SPEED_. These results indicate once again that whether or not RT_SPEED_ is taken into account in IIV_RT_ analysis can affect study outcome and that this effect can vary with respect to the groups investigated and the processing demands of the task.

Raising the processing load increased the group difference in RT_SPEED_ between aMCI^+^ and CH (from effect size, Cohen’s *d* = .78 to.9) and reduced the comparison of RT_SPEED_ between AD and CH (from effect size, Cohen’s *d* = 1.62 to 1.4). For IIV_RT_ increasing the processing load resulted in a reduction in the effect size between aMCI^+^ and CH (from effect size, Cohen’s *d* = .63 to.52) compared to the low processing load condition and also only slightly reduced the differentiation in IIV_RT_ between AD and CH (from effect, Cohen’s *d* = 1.3 to 1.2). Therefore increasing the processing load of a task per se does not necessarily increase group differentiation in the study of MCI and AD, appearing instead to be determined by factors such as what is being measured (e.g. RT_SPEED_ or IIV_RT_), the group under study and indeed whether or not RT_SPEED_ is taken into account in IIV_RT_ analysis.

In CH, the absence of gender related influences upon RT_SPEED_ and IIV_RT_ in the low- processing load condition was maintained when processing load increased. However, in the aMCI^+^ group, the slower RT_SPEED_ and greater IIV_RT_ for female compared to male patients in the low processing load condition were abolished with the increase in processing load. Thus gender effects also appear contingent upon the task employed. Although we were unable to examine gender-related effects in our AD group the possibility arises that gender may also influence research in this group of people.

### Study Limitations

As already highlighted, it is possible that in our study of IIV_RT_ and RT_SPEED_ in aMCI^+^ compared to CH, outcome was affected by the proportion of aMCI^+^ patients with pro-dromal dementia. In the absence of longitudinal analysis, we cannot determine whether, for example, this affects the magnitude of the effect between CH and aMCI^+^ per se and especially whether the significantly greater IIV_RT_ and RT_SPEED_ for female compared to male patients may be simply a result of the fact that a greater proportion of females had pro-dromal dementia. The lack of white matter analysis (e.g. DTI) or other functional/anatomical imaging techniques also precluded the investigation of the relationship between structural and functional brain changes, IIV_RT_, RT_SPEED_, cognition, cognitive reserve and gender. In the study of RT_SPEED_ and IIV_RT_ there are many factors in addition to the ones investigated here, potentially capable of influencing both the speed and variability of processing in ageing, aMCI^+^ and AD and thus warrant further investigation. Such factors include fatigue and practice effects, stimulus characteristics, sensory-motor integration, decision and response and temporal factors, e.g., [94]. Finally, a larger sample size would more confidently confirm differences and would have permitted also the potentially more clinically appropriate comparison of female patients with female controls and male patients with male controls.

### Summary

We have shown that in the study of IIV_RT_ in CH, MCI and AD, study outcome is prone to influence by a variety of factors acting independently and possibly interactively. This evidence of raised IIV_RT_ and slowed RT_SPEED_ also indicates that information processing in MCI and AD may be more compromised than revealed by routine neuropsychological testing and so may impact upon daily behaviours which depend upon RT_SPEED_ and consistency of processing, such as driving and avoidance of falls.
